# Supplementary material for: Trends and forecasts of leprosy for a hyperendemic city from Brazil’s northeast: Evidence from an eleven-year time-series analysis
Source: PLoS One. 2020 Aug 7;15(8):e0237165. doi: 10.1371/journal.pone.0237165 (PMC7413479; doi:10.1371/journal.pone.0237165)
Supplement: Attachment — Submitted filename: Response to Reviewers.pdf [file pone.0237165.s005.pdf]

Dear Editor,

Many thanks for your reply and your reviewers' comments about our manuscript **"PONE-D-20-00967 Trends and forecasts of leprosy for a hyperendemic city from Brazil's northeast: Evidence from an eleven-year time-series analysis".**

The comments were appropriate to qualify/ improve the manuscript. We have forwarded a letter with the changes made in the manuscript, presenting our response for each comment from reviewers. Many thanks for your comments.

Kind regards,

Ramos et al.

Editor comments:

After final review, I find the manuscript has resolved most of the questions that were addressed to the authors. In the rewrite, there are some small edits that should be considered for revision:

Line 63 change a to the

Authors: The term was included according to the suggestion.

Lines 61 – 64: "After the introduction of Multidrug Therapy (MDT) and the high vaccination coverage of the Bacillus Calmette-Guérin (BCG), especially in children, the burden of leprosy has decreased considerably worldwide".

Line 68 remove : after are and remove ; and number

Authors: Terms have been revised.

Lines 67-72: "In 2016, the World Health Organization (WHO) published the Global Leprosy Strategy 2016−2020: Accelerating towards a leprosy-free world, the objectives are to decrease the disease's global and local burden, decrease the cases of children with deformities, decrease the new cases diagnosed with grade 2 physical disabilities to less than one case per 1 million

inhabitants, and review all laws that somehow lead to the discrimination against people with leprosy [4].”

Line 167 – do you mean causal and not casual?

Authors: We mean causal. The sentence were corrected.

Lines 165-167: “Leprosy detection rates were smoothed by the moving average technique, considering the average of three months (prior, current and posterior), in order to remove noise and better reveal the underlying causal process”.

Line 292 change to high rates of disease were found

Authors: The sentence were corrected

Lines: 291-292: “Despite the downward trend seen in the age group between 30 and 59 years old, both among women and men, high rates of disease were found in the entire study period”.

Line 369 change most of the reported cases were male to largest proportion of cases were male

Authors: The sentence were corrected

Lines 368-370: “In this context, largest proportion of cases were male, which is in agreement with data provided in the literature, in which those affected by the disease in most world regions are predominately male, including in Brazil [27–29]”.

Line 374 punctually is not clear and I would suggest rewording

Authors: The word was reformulated according to the suggestion.

Lines 373-374: “In a few periods of the time series, women had slightly higher detection rates than men”.

Line 381 this is a long paragraph, suggest two sentences

Authors: The authors agree with the editor. The paragraph was divided into two sentences.

Lines 381-386: “Other factors may also explain the higher occurrence of the disease in men, as individual determinants, such as not seeking medical assistance or only later seeking health services, when compared to the practices of women. There are also operational issues concerning difficulties accessing health services in a timely manner due to an incompatibility between the

men’s and the units’ working hours, a lack of a health policies directed to men, and restricted access to health information [15,16]”.

Line 389 I don’t see where transmission is increasing in men from the data, I’m not sure if I’m missing something. Transmission could be continuous because of maintenance of the disease but I don’t see where in the data you are indicating increases in this group

Authors: We agree with the comment. According to results it is not possible to confirm that transmission is increasing in men. We make it clear that from the results we have strong indications that transmission is greater in men.

Lines 387-389: “The higher detection rates in men compared to women, in the research scenario, especially in the period from 2011 to 2016, are strong indications that transmission is higher in men”.

Lines 397 and 401 change to year not years

Authors: The sentence was reformulated according to the suggestion.

396-403: “In terms of age, most of the individuals affected are aged between 30 and 59 years old, followed by the 15 to 29 year old group, though the population over 59 years old presents the highest detection rates.

Despite the high rates in the 30 to 59 year age group, it should be noted that the temporal trends in these intervals were decreasing in the study period. And the 15 to 29 year old interval also showed the highest average percentage decrease in the period from 2006 to 2016, which points to an indication of a reduction in bacillus transmission in the studied region [32,35]”.

Line 404-407 this is a run on sentence, suggest revision

Authors: The sentence was revised.

Lines: 404-406: The group aged between 30 and 59 years old includes the Brazilian economically active population, interval that the disease hinders labor activities, forcing individuals to stop working or retire early, decreasing the quality of life of workers [28,36,37].

Line 408 remove “reaching”; change diagnoses to diagnosis of cases; in addition to… suggest to reword for clarity

Authors: The review was carried out according to the suggestions

Lines 406-410: “In this sense, health services should focus on preventive measures by actively seeking individuals in this age group, in addition to diagnosis of cases, providing timely

treatment, and early identification of lesions, the purpose of which is also to prevent physical disability”.

Line 411 change “due to” to from

Authors: The sentence were corrected

Lines 410-412: “Early interventions prevent or minimize the high social costs leprosy imposes from removing this population from productive activities and social relationships [28,36-38]”.

Line 418 change to population and remove the apostrophe

Authors: The sentence were corrected.

Lines 417-421: “Considering the rapid aging process of the Brazilian and Imperatriz population, when leprosy is diagnosed and treated late, it leads to the functional loss of peripheral nerves and physical impairment, which, combined with the aging process and other comorbidities, contribute to elderly individuals’ greater vulnerability and loss of autonomy [41]”.

Line 424 and paragraph – this is not clear – you state that there is a continuous increase in the rate of detection that indicates a decrease in transmission. Are you intending to show that because the increase is in the older population and other ages show a decline, that this is an indicator in overall reduction? If so, you may want to revise. Although you do also show increases in <15 years among males. Alternatively you could remove the sentence since you have described the decreasing tendency in the following paragraph, and include with the exception of women 60 and older and males <15

Authors: In line 424 and in the paragraph we intended to show that the increase in the detection of elderly people may be indicative of a change in the epidemiological profile of the disease.

In some countries, the elimination of leprosy was followed by a change in the profile of the disease, with the greatest detections in the elderly.

Despite this change in the leprosy profile, Imperatriz has hyperendemicity of the disease.

We agree with the editor's suggestion and rewrite the paragraph, we try to make the paragraph more clearer. Thank you very much for the suggestion for improvement.

Lines 422-430: “Countries that registered a decrease in leprosy transmission, with subsequent elimination, observed a change in the profile of the disease, with a drop in detection in younger age groups and an increase in detection of elderly people [35,42]. In Imperatriz, the high detection rates in the population aged 60 years old or over may be an indicative of a change in the epidemiological profile of the disease, despite the municipality still showing levels of hyperendemicity.

A decreasing tendency was found in the total detection rates of both men and women from 2006 to 2016, however, considering the age groups, women aged 60 years old or over and men aged less than 15 years old showed an increasing trend".

Line 438 include a period (.) after trends for a new sentence

Authors: Inclusion carried out.

Line 437: "This study's findings show total and by-sex downward trends".

Line 432 you discuss improvements in the decline of cases due to improved control efforts. However line 446 indicates that the process is weak. Perhaps reword to indicate that the process could be improved upon

Authors: According to the suggestion, we indicate on line 446 that the process can be improved.

Lines 444-446: "Potential explanations include difficulty establishing a clinical diagnosis, disease-related stigma, and the weak health promotion and education process, needing improvements in leprosy control actions in these areas [15,45,46]".

Line 448 include For example at the start of the sentence. Change to an assessment of health services

Authors: The sentences were corrected according to the suggestion.

Lines 447-449: "For example, studies conducted in two Brazilian regions in which leprosy is endemic an assessment of health services identified that the local primary health units did not present satisfactory performance in diagnosing individuals younger than 15 years old".

Line 451 reword "passively assisted voluntary demand" to health diagnosis was conducted on request, however active case detection in the community is not being conducted

Authors: The sentence was reformulated according to the suggestion.

Lines 450-451: "The reason for this is that the services health diagnosis was conducted on request, however active case detection in the community is not being conducted. [46,47]".

Line 464 long sentence, suggest revision

Authors: The sentence has been reformulated. It was divided into two sentences.

Lines 463-468: Although the present study does not measure the impact of this action, the hypothesis arises that the National Campaign for Leprosy may be influencing the detection of cases under the age of 15 years old in the municipality, especially due to the rates found in the investigated period. It should be noted that the beginning of the campaign (2013) is concomitant with the beginning of the growth trend in men under 15 years old (2014)".

Line 486 revise to proposed the inclusion of

Authors: The sentence was revised.

Lines 483-487: "The Enhanced Global Strategy for Further Reducing the Disease Burden Due to Leprosy (2011-2015) from WHO proposed the inclusion of female leprosy cases indicator among the total number of new cases, in order to assess and ensure that women are having adequate access to leprosy diagnostic services [54]".

Line 487 revise to total number

Authors: Revised.

Lines 483-487: "The Enhanced Global Strategy for Further Reducing the Disease Burden Due to Leprosy (2011-2015) from WHO proposed the inclusion of female leprosy cases indicator among the total number of new cases, in order to assess and ensure that women are having adequate access to leprosy diagnostic services [54]".

Line 549 do you mean database here?

Authors: Yes, it is a database. The term was corrected.

Lines 548-550: "This study's limitations include the fact a secondary database was used, with inconsistent quality and quantity of information, with the potential presence of ignored or incomplete data".

Reviewers' comments:

Reviewer #1: (No Response)

Reviewer #2: The manuscript reads much better now.

I would like to make few additional suggestions:

Abstract

Line 40 – I suggest using "average incidence" instead of "incidence" only

Authors: The term was included according to the suggestion.

Lines 39-42: "A total of 3,212 cases of leprosy were identified, the average incidence among men aged between 30 and 59 years old was 201.55/100,000 inhabitants and among women in the same age group was 135.28/100,000 inhabitants".

Line 48 – I suggest saying that 'the city is unlikely to meet a "significant decrease" of the disease burden by 2020'

Authors: The reviewer's suggestion was considered.

Lines 46-48: "Even though the forecasts show a downward trend in Imperatriz, the city is unlikely to meet a significant decrease of the disease burden by 2020".

Introduction

Line 75 – I suggest using "In the same year" instead of "In the same period"
Authors: The term was included according to the suggestion.

Lines 75-77: "In the same year, Brazil presented a detection rate of new cases of 12.94 cases/100,000 inhabitants, accounting for 93% of the total cases reported in the Americas [5,6]".

Materials and methods

Line 134 – "In the same period" means 2010? If so, it would be better to use "the same year". The same should be checked for line 140.
Authors: The periods refers to 2010. We correct for "the same year".

Lines 134-140: "In the same year, the main social indicators are: an illiteracy rate of 9.7%, Human Development Index (HDI) of 0.73 and Gini Index of 0.46. In terms of basic sanitation, 23% of the city has a sewage system and 86% has a drinking water supply [18,19].

In 2016, the detection rate of new cases of leprosy in the state of Maranhão was 47.30 cases/100,000 inhabitants, classifying the state as the third most endemic in Brazil. In the same year, Imperatriz presented a detection rate of 62.23 cases/100,000 inhabitants, marking it as a Brazilian city with hyperendemicity levels [6,9,20]".

Results

Line 237 – Revise the use of "both" once you report results for 3 groups, or rephrase it using two categories (for example: "total of cases and cases by sex")

Authors: The excerpt was revised according to the suggestion.

Lines 235-238: "Table 1 presents the descriptive statistics of cases according to sex, age groups and AMPC, showing in absolute numbers, that the group aged between 30 and 59 years old predominated among total of cases (1566; rate=166.46/100,000 inhabitants), men (892; rate=201.55/100,000 inhabitants) and women (674; rate=135.28/100,000 inhabitants)".

Discussion

Line 429 – Replace "by" for "be"

Authors: The term was replaced according to the suggestion.

Lines 424-427: "In Imperatriz, the high detection rates in the population aged 60 years old or over may be an indicative of a decrease in transmission, despite the municipality still showing levels of hyperendemicity".

Lines 457-461 – Authors have just stated (lines 452-456) that primary health units do not present satisfactory performance in diagnosing leprosy in children because they don't actively seek cases. Thus it is surprising that Anchieta's study (2019) observed a decreasing trend in the detection of children under 15 years old because of active case-finding campaigns in the school-aged population. These two statements are not in agreement with each other.

Authors: The studies on lines 448-452 state that their scenarios did not perform satisfactory for the diagnosis of leprosy in children because local health units do not active case-finding. These studies [46,47] assess the performance of local health units. These studies were carried out before the year 2013, before the National Campaign for Leprosy, Vermin, Trachoma and Schistosomiasis

In the study by Anchieta, conducted in 2019, the consequences of national campaigns of active case-finding in school-aged children were discussed, which contributed to the drop in the trend of detecting children under 15 years of age.

We emphasize that the studies [46, 47] are local, and that Anchieta's study refers to national campaigns.

Lines 447-456: "For example, studies conducted in two Brazilian regions in which leprosy is endemic an assessment of health services identified that the local primary health units did not present satisfactory performance in diagnosing individuals younger than 15 years old. The reason

for this is that the services health diagnosis was conducted on request, however active case detection in the community is not being conducted. [46,47].

Anchieta et al. (2019) [43] identified that Imperatriz showed a decreasing trend in the detection rate of children under 15 years old in the period from 2001 to 2015, a phenomenon explained by active case-finding national campaigns in the school-aged population in the years 2013 and 2016. Despite this decrease, the authors reaffirm that detection in children under 15 years old remains high in the municipality".

Line 477 – We suggest to include "leprosy" before "patients"

Authors: The term has been included.

Lines 471-474: "According to Monteiro et al. (2013) [36], 60.3% of the leprosy patients located in the north of Brazil were male individuals aged over 60 years old, while Nobre et al. (2017) [32] determined that 15.11% more men than women were affected by the disease".

Line 552 – Please replace "eliminate of transmission" by "eliminate the transmission"

Authors: The term was replaced according to the suggestion.

Lines 545-547: However, considering the forecasts and trends, leprosy will remain endemic and the WHO global goals to decrease the disease's burden and eliminate the transmission of leprosy by 2020 may not be met.
