# Supplementary material for: Trends and forecasts of leprosy for a hyperendemic city from Brazil’s northeast: Evidence from an eleven-year time-series analysis
Source: PLoS One. 2020 Aug 7;15(8):e0237165. doi: 10.1371/journal.pone.0237165 (PMC7413479; doi:10.1371/journal.pone.0237165)
Supplement: Attachment — Submitted filename: Second revision comments.docx [file pone.0237165.s004.docx]

After final review, I find the manuscript has resolved most of the questions that were addressed to the authors. In the rewrite, there are some small edits that should be considered for revision:

- Line 63 change a to the
- Line 68 remove : after are and remove ; and number
- Line 167 – do you mean causal and not casual?
- Line 292 change to high rates of disease were found
- Line 369 change most of the reported cases were male to largest proportion of cases were male
- Line 374 punctually is not clear and I would suggest rewording
- Line 381 this is a long paragraph, suggest two sentences
- Line 389 I don’t see where transmission is increasing in men from the data, I’m not sure if I’m missing something. Transmission could be continuous because of maintenance of the disease but I don’t see where in the data you are indicating increases in this group
- Lines 397 and 401 change to year not years
- Line 404-407 this is a run on sentence, suggest revision
- Line 408 remove “reaching”; change diagnoses to diagnosis of cases; in addition to… suggest to reword for clarity
- Line 411 change “due to” to from
- Line 418 change to population and remove the apostrophe
- Line 424 and paragraph – this is not clear – you state that there is a continuous increase in the rate of detection that indicates a decrease in transmission. Are you intending to show that because the increase is in the older population and other ages show a decline, that this is an indicator in overall reduction? If so, you may want to revise. Although you do also show increases in <15 years among males. Alternatively you could remove the sentence since you have described the decreasing tendency in the following paragraph, and include with the exception of women 60 and older and males <15
- Line 438 include a period (.) after trends for a new sentence
- Line 432 you discuss improvements in the decline of cases due to improved control efforts. However line 446 indicates that the process is weak. Perhaps reword to indicate that the process could be improved upon
- Line 448 include For example at the start of the sentence. Change to an assessment of health services
- Line 451 reword “passively assisted voluntary demand” to health diagnosis was conducted on request, however active case detection in the community is not being conducted
- Line 464 long sentence, suggest revision
- Line 486 revise to proposed the inclusion of
- Line 487 revise to total number
- Line 549 do you mean database here?
